# Developing Ubiquitous Sensor Network Platform Using Internet of Things: Application in Precision Agriculture

**DOI:** 10.3390/s16071141

**Published:** 2016-07-22

**Authors:** Francisco Javier Ferrández-Pastor, Juan Manuel García-Chamizo, Mario Nieto-Hidalgo, Jerónimo Mora-Pascual, José Mora-Martínez

**Affiliations:** Department of Computer Technology, University of Alicante, P.O. Box 99, E-03080 Alicante, Spain; juanma@dtic.ua.es (J.M.G.-C.); mnieto@dtic.ua.es (M.N.-H.); jeronimo@dtic.ua.es (J.M.-P.); jose.mora.martinez.es@gmail.com (J.M.-M.)

**Keywords:** precision agriculture, ubiquitous sensor network, internet of things

## Abstract

The application of Information Technologies into Precision Agriculture methods has clear benefits. Precision Agriculture optimises production efficiency, increases quality, minimises environmental impact and reduces the use of resources (energy, water); however, there are different barriers that have delayed its wide development. Some of these main barriers are expensive equipment, the difficulty to operate and maintain and the standard for sensor networks are still under development. Nowadays, new technological development in embedded devices (hardware and communication protocols), the evolution of Internet technologies (Internet of Things) and ubiquitous computing (Ubiquitous Sensor Networks) allow developing less expensive systems, easier to control, install and maintain, using standard protocols with low-power consumption. This work develops and test a low-cost sensor/actuator network platform, based in Internet of Things, integrating machine-to-machine and human-machine-interface protocols. Edge computing uses this multi-protocol approach to develop control processes on Precision Agriculture scenarios. A greenhouse with hydroponic crop production was developed and tested using Ubiquitous Sensor Network monitoring and edge control on Internet of Things paradigm. The experimental results showed that the Internet technologies and Smart Object Communication Patterns can be combined to encourage development of Precision Agriculture. They demonstrated added benefits (cost, energy, smart developing, acceptance by agricultural specialists) when a project is launched.

## 1. Introduction

The integration of information and control technologies in agriculture processes is known as Precision Agriculture (PA). In order to obtain the greatest optimization and profitability PA adapts common farming techniques to the specific conditions of each point of the crop, by applying different technologies: micro-electro-mechanical Systems, Wireless Sensor Networks (WSN), computer systems and enhanced machinery [1]. The right application of this method guarantees satisfactory results [2]. These technologies can be implemented, among others, in indoor, outdoor and hydroponics [3] crops; and its management is mainly divided into three stages [4]. The first one is called determination stage, wherein the crop type is identified and areas thereof are clustered based on their homogeneity. In this stage, sensors are the devices that analyse the characteristics of each area. The collected data is processed in the second stage (analysis) by computer systems, which establishes procedures to apply in each area. Finally, the execution stage is carried out by the advanced machinery.

**Figure 1:**
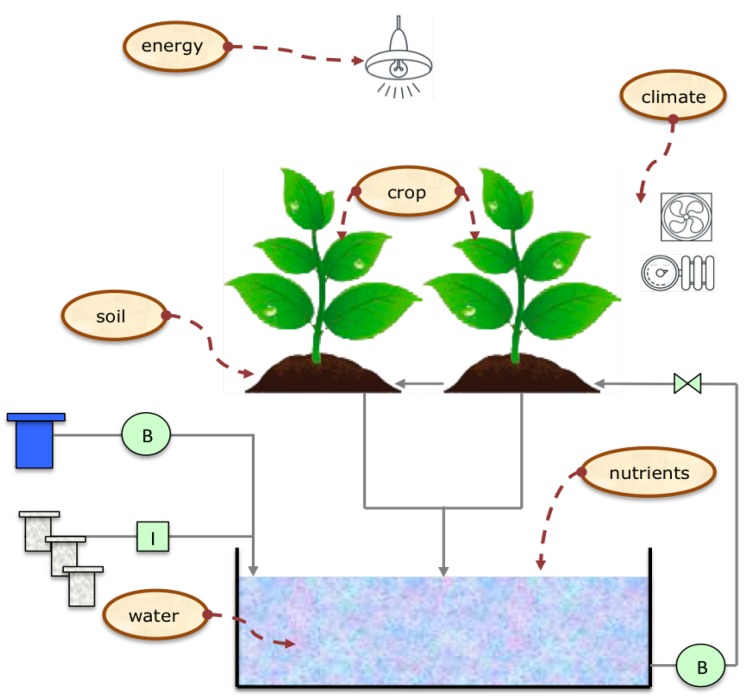
Agricultural Production Subsystems.

In the past few years, new trends have emerged in the agricultural sector. Thanks to developments in the field of WSN as well as miniaturization of the sensor boards, PA has started to emerge [5,6,7]. However, even though there has been considerable progresses in different technological areas, few of them are focused on the design and implementation of specialized low-cost systems for agricultural environments [8]. Traditionally, information technologies are available but they have not been widely introduced in agricultural scenarios: expensive systems and difficulties in installing, controlling, and maintaining these installations are being the main barriers. In this regard, new platforms particularly directed to remove these barriers are needed. This work proposes a solution based on an heterogeneous [9] and scalable platform, able to acquire, process, store and monitor data from crop growing systems using a mobile ubiquitous approach. This approach is developed with Ubiquitous Sensor Networks (USN) and Internet of Things (IoT) paradigms. The platform is specialized in crop lands automation maintenance, which aim to control the conditions that determine the proper development of a crop, such as substrate and atmospheric temperature, luminance, atmospheric humidity, water PH level, Electric Conductivity (EC); and the actuators management which influence these factors. The agricultural processes treated are based in six subsystems (Figure 1): crop, soil, water, nutrients, climate and energy model.

This paper is organized as follows: Section 2 reviews related works. Information Technologies applied in PA are reviewed. Section 3, platform specifications and requirements in PA scenarios are analysed. A design method is proposed to develop a platform which uses USN architecture in agricultural production and IoT technologies for the development of effective services. Section 4, experiments including a prototype development based in an hydroponic installation on site are analysed and discussed to assess suitability of this method. Finally, Section 5 provides results and conclusions.

## 2. Related Work

PA observes, measures and responds to inter and intra-field variability in crops. The obtained benefits are mainly increased production and profitability. Other benefits come from better working conditions. Thus, PA contributes to the wider goal concerning sustainability of agricultural production. The implementation of PA has become possible thanks to the development of sensor technologies combined with procedures to link mapped variables to appropriate farming management actions such as cultivation, seeding, fertilization, herbicide application, and harvesting. Many sensors are currently available and used for data gathering or information provision as part of the PA implementation. These devices are designed for both in situ and on-the-go recording. Devices exist to assess the status of soils, such as apparent EC sensors, gamma-radiometric soil sensors, and soil moisture devices, among others. Others record weather information or micro-climate data (thermometer, hygrometer, etc.). Particular importance is given to sensors that quantify the physiological status of crops (e.g., Nitrogen sensors) [10].

Sensors have traditionally been essential elements in industrial processes due to their ability to monitor the physical parameters involved in the different production processes. Connectivity between them was done using traditional wired networks.

It is accepted that PA provides benefits, however a number of factors may slow down its integration:

- **Initial expectations and advantages promised unfulfilled.** The farming community strongly endorse the adoption of PA technology to manage variability within paddocks. Nevertheless they have become frustrated with the technology, lack of support mainly, and this is the main impediment for PA to be adopted [11]. At a practical level, farmers need to gain the confidence in how to deal with variability and work in partnership with PA specialists
- **Complexity of technology and incompatibility of components**. The rate of adoption of PA is expected to continue to rise based on greater awareness of the benefits of the technology. There are constraints to adapt the technical issues with equipment and software and the incompatibility of equipment with existing farm operations [12].
- **Lack of products**. The PA integration has been delayed, in part, due to the lack of products that bring together engineering and agronomics. In [13] this situation has been presented. One solution (proposed in this paper) is to integrate data collection, data processing, and management actions.
- **High costs in start-up and maintenance of the facility**. There are high start-up and maintenance costs in PA adoption, in some cases with a risk of insufficient return on the investment. In [14], German farmers were interviewed about their experiences with PA technology and their attitudes and barriers towards it, the majority of the interviewed farmers hesitated to introduce PA techniques mainly because of the high costs of the technology.

Currently, continuous technological advances have spurred the development of devices with wireless communication capabilities, arranged at any location, getting smaller and smaller, autonomous, more powerful and with a more efficient battery consumption [15]; which communicate the collected data to a centralized processing station. This is important since many network applications require hundreds or even thousand of sensor nodes, often deployed in remote and inaccessible areas. Therefore, a wireless sensor has not only a sensing component, but also on-board processing, communication, and storage capabilities. With these enhancements, a sensor node is often not only responsible for data collection but also for in-network analysis correlation, and fuses its own sensor data from other sensor nodes. When many sensors monitor large physical environments cooperatively, they form a WSN [16]. Sensor nodes communicate not only with each other but also with a base station, allowing them to disseminate their data to remote processing, visualization, analysis and storage systems [17].

There are some applications based on the IoT paradigm that may be available on our smart phones and help in agricultural areas to control crops. One of the most promising market segments in the future, is precisely the use of identifiable wireless devices in applications related to nature and environmental preservation [18].

The development of WSN applications in PA makes possible to increase efficiency, productivity and profitability in many agricultural production systems, while minimizing unintended impacts on wildlife and the environment. The real time information obtained from the fields can provide a solid base for farmers to adjust strategies at any time. Instead of making decisions based in some hypothetical average conditions, which may not exist actually anywhere, a precision farming approach recognizes differences and adjusts management actions accordingly [19].

The combination of WSN, which are cheaper to implement than wired networks [20], with intelligent embedded systems and applying on this combination the technology of ubiquitous systems [21], leads to the development of the design and implementation of low cost systems for monitoring agricultural environments, which due to its low cost, are optimized for developing countries and difficult access areas. The state of the art shows different problems which need to be resolved to achieve full integration of control and information technologies in normal activities carried out on agriculture.

### 2.1. IoT Precision Agriculture

Initial work is shown in the previous subsection. Different proposals take the conventional Internet paradigms (web services) and some WSN protocol (Zigbee, Bluetooth, Z-Wave, etc.) to built new services and platforms. Monitoring applications and cloud services (analysis and statistic) are the main advantages. In Table 1 main characteristics are shown analysing different scientific works and industrial platforms in PA scenarios.

### 2.2. First Conclusions

The first conclusions are:

- Current proposals use conventional automated systems on web services.
- Different PA IoT models are proposed using three levels: control, communication and application layer (cloud services). These models have yet to be put into practice and tested with real applications.
- Agricultural production controlled by agronomist and methods to develop low-cost prototypes are not available.
- Response time controlled on multi-protocol integration is unusual.
- Industrial applications are available. High costs, and the high-level of expertise required for controlling and maintaining are some of the characteristics of these applications.

The full potential of these technologies can incorporate new features and improvements which are as yet undeveloped in real scenarios.

Response time communication for automated processes. Response time controlled using IoT sensor/actuator nodes could be distributed in different networks (USN) making all devices interoperable in control applications. All control devices can use transparently any sensor/actuator in their control processes. Machine-to-machine (M2M) applications and human machine interface (HMI) could be integrated and adapted to the needs of the users and specific production. M2M will be used on automated processes and HMI will be used by agronomists on data monitoring, programming events with IF...THEN rules and some manual control actions. Methods to develop low cost prototypes could be designed, easily tested and adapted by agronomist in real agriculture production.

Response time controlled and interoperable sensor/actuator nodes are developed using edge computing and machine-to-machine (M2M) protocol. With edge computing, a local application layer is developed to create control rules and process data. With M2M protocol all nodes are interoperable and edge computing processes can use them.

## 3. Network Platform: Design and Development

The Internet Network was originally designed in a client-server model, where the client was always the initiator of the request. Devices initiate the communication whenever they need to push data to the cloud. In IoT applications the server needs to push data to a client without the client first making a request. Web developers have come up with some techniques to overcome this challenge. Here are three options that we consider more portable to the embedded world:

- **Short polling**: method where the client periodically connect the server (new data are available to communicate). This is the simplest solution to code. It is not recommended in real time.
- **Long polling**: the client performs the request and the server do not respond until it has something to send. This enables real-time push notifications from the cloud to devices. This technique consumes more energy and also risks the loss of the connection.
- **Adapted protocols**: to use newer protocols like Constrained Application Protocol (CoAp) or Message Queue Telemetry Transport (MQTT), designed to provide low latency, small packet sizes and stable communication over weak networks. These protocols provide a two-way communication channel, which in turn supports push notifications. This makes them good choices for IoT projects requiring the ability to control connected devices in real time. Firmware libraries and examples for embedded devices, normally using HTTP-based connections, are required for developing new applications.

Choosing the right protocol depends on IoT application and how much time is needed to communicate with the devices. Short polling can be favourable in projects where the data needs to be updated in specific periods of time. Adapted protocols are optimised for control and two-way open communication channels. Long polling can be useful in some services of notifications.

In this work MQTT protocol is proposed as communication paradigm between sensor/actuator devices. Some of the features that makes it especially suitable for this project are:

- MQTT is a publish-subscribe messaging protocol developed for resource-constrained devices, a model already in use by enterprises worldwide, and can work with legacy systems.
- All messages have a topic path composed of words separated by slashes. A common form is /place/device-type/device-id/measurement-type/status. The subscribers may use wild-cards to subscribe to all measurements coming from a specific class of device.
- The bandwidth requirements are extremely low, and the nature of the protocol makes it very energy-efficient.
- Defunct nodes are easily discovered: A publisher can register a Â«last will and testamentÂ» which the MQTT server sends to the subscribers of a topic after a certain time-out.
- The programming interface is very simple, and the client memory footprint is small, making it especially suitable for embedded devices.
- Three Quality of Service (QoS) levels provide reliable operations.

**Figure 2:**
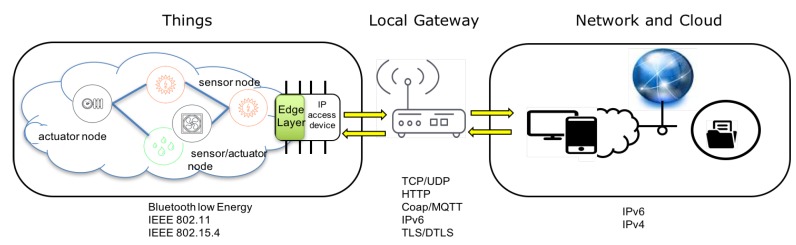
IoT model used: device-to-gateway pattern (RFC7452).

Ubiquitous networks allow an *n-to-m* nodes communication model (Figure 2). Any node is able to query and be queried by other nodes. In addition, any node may play the role of a base station (skin node) capable of transmitting its information to remote processing places using a gateway device. USN local nodes can use and process local data, with a gateway these nodes have a global accessibility and they offer extended services on an IoT scenario. Local and global access over the same node (sensor/device/actuator) has different possibilities and benefits. Whereas a local data processing is necessary in basic process control (security, system start-stop, etc.), global processing (analytic) can be used in pattern detection and information generation. In this sense, the proposed platform uses both technologies combined: different USN over a local network area (intranet) connected to cloud-IoT services (internet). A computing layer in local area, called *edge computing*, will serve as interface between control processes and cloud-services. This layer will be able to process data before communicating to cloud.

### 3.1. Platform Design: Analysis and Requirements

Agriculture is a complex system, related to a wide range of environments, which is difficult to deal perfectly. In all natural or agricultural systems, a hierarchy of levels exist [31]. For irrigated crop production this hierarchy might be: plant, cropping systems, farming and drainage systems.

Each agricultural subsystem is composed by things (sensor/actuator) that can be connected and processed using USN and IoT paradigms. The requirements established on the IoT platform for PA scenarios are:

1. Low-cost elements and low-power consumption of all devices installed improve the establishment and development.
2. Standard communication protocols to develop open and reusable applications.
3. Easy access and maintenance. Users are not specialized in information technologies; this can improve its acceptability.
4. Support the integration of new smart control modules (modularity and scalability).
5. To ensure interoperability when new devices are required.
6. Non-proprietary hardware-software to reduce dependences.

The platform communication follows a pattern based in an IoT model: Device-to-gateway (RFC7452 [32]). Over this pattern, edge layer computing is developed. The platform is based in three elements:

- **Things**. Today there are millions of things (sensors/actuators and devices found in commercial and industrial settings) connected directly through wireless networks and accessing the Internet. Usually, the IoT solutions have things filtered and managed using data locally and/or connected to gateways that provide extended functionality. Basic devices are tagged like things. Each thing has data that can be shared in the Internet.
- **Local Gateway**. Most of existing things were not designed to connect to the Internet and cannot share data with the cloud. To resolve this difficulty gateways act as intermediaries between things and the cloud, providing the needed connectivity, security, and manageability.
- **Network and Cloud**. Cloud infrastructure contains large pools of virtualized servers and storage that are networked together. IoT solutions run applications that analyse and manage data from devices and sensors in order to generate services that produce information used in decision making.

The device-to-gateway communication pattern can frequently be found with smart object deployments that require remote configuration capabilities and real time interactions. The gateway is thereby assumed to be always connected to the Internet.

However, the gateway can be mobile, i.e., a smart-phone. In these situations connectivity between the devices and the Internet may be intermittent. This limits the applicability of such a communication pattern but is very common with wearable and other IoT devices that do not need always-on Internet. From an interoperability point of view, it is worth noting that smartphones, with their sophisticated software update mechanism via app stores, allow new functionality to be updated regularly at the smartphone and sometimes even at the IoT device. With special *apps* that are tailored to each specific IoT device, interoperability is mainly a concerned to the lower layers of the protocol stack, such as the radio interface, and less so at the application layer.

### 3.2. Platform Development

**Figure 3:**
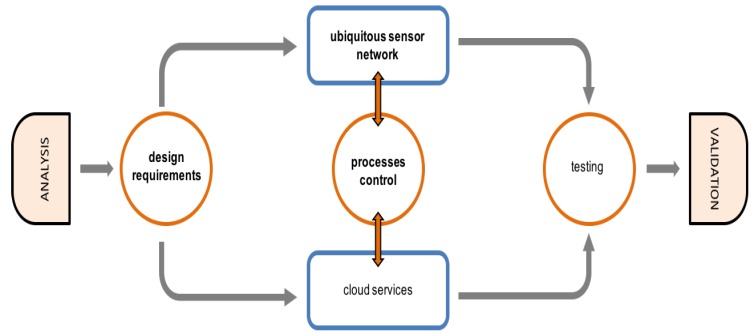
Design and development: project planning.

In order to build a monitoring and control installation on PA scenarios, design recommendations should be considered. The flow diagram in Figure 3 is the reference model proposed to develop this type of installations, using IoT paradigm. Agricultural subsystems (crop, soil, water, etc.) are associated with this model (analysis, control, etc.) to determine development requirements. Things (sensor/actuators and devices) are inter-operable and are installed in an ecosystem based in ubiquitous sensor network that use IoT paradigms to communicate and to create cloud services.

**Figure 4:**
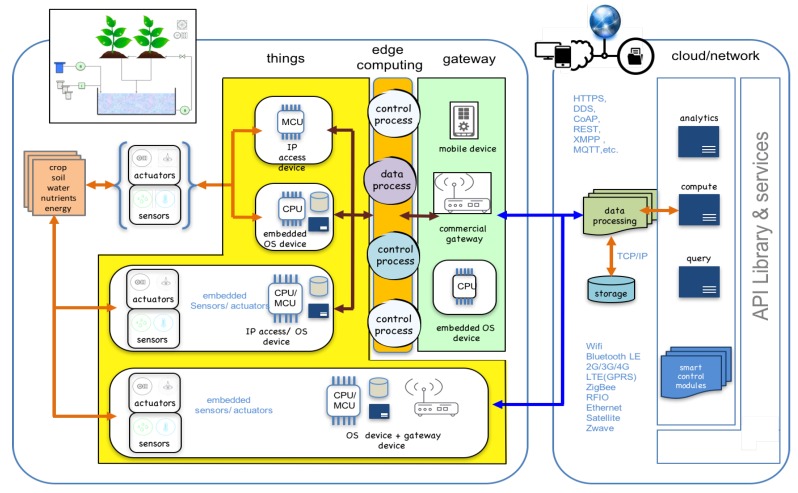
IoT ecosystem developed. Sensors, actuators and IP access devices make up the ubiquitous thing layer.

The ecosystem proposed is shown in Figure 4. Following platform design model in the IoT ecosystem, PA processes are developed following different stages:

- **Analysis**: agronomist and Information and Communication Technologies (ICT) technicians should set the basic goals for the project. Scenarios, objectives and technical needs are identified in this phase.
- **Design requirements**: there are requirements already established on the IoT platform (low-cost, interoperability, standard communication protocols, easy access and maintenance, modularity and scalability and non-proprietary dependences). In this phase, these requirements are related with the requirements of the agricultural installation analysed. A design based in things and its requirements (compute and communication) is proposed by agricultural and ICT technicians. Things and agricultural processes are defined.
- **Ubiquitous sensor network**: sensors/actuators (things) and its communication/processing capabilities should be clearly defined. Also, the intermediary (network access nodes) collecting information from a group of things must be proposed. These nodes facilitate the USN network access, communicating with a control centre or with external entities. In this phase the architecture of communication layer is proposed.
- **Control Processes**: agricultural activities with basic and advanced rules between things are specified by agronomists. A technology platform (middleware) to enable the effective use of a USN in the particular application is proposed by ICT technicians. Control rules and things relationships are modelled.
- **Edge computing**: enables analytic and knowledge generation at the source of the data. In this work, Edge is the layer between data sources and cloud data centers where M2M protocol, control, data processing and web services are integrated. Edge computing pushes applications, data and processes away from embedded nodes that control USN to the logical extremes of a cloud network.
- **Cloud services**: monitoring, processing and analytic services, among others, can be designed in this phase using Internet technologies. Query and analytic layers are also defined. Things and services are transparent because both cloud and stand-alone cloud use the same paradigms, technologies and protocols.
- **Test and validation**: things, control processes, USN, Internet communication and Cloud applications are tested and validated in real scenarios.

### 3.3. Platform Architecture

Services and activities can be built and distributed in modules localised in local, mobile or cloud storages. Moreover, things can be used in local installation (sensors, actuators, control devices) or in mobility (smart-phones, embedded devices). Considering the above, the architecture proposed is composed by distributed modules (sensors or actuators groups, processes, virtual services, storage, analytic, etc.) on a basic structure of four layers: Things, Edge, Communication and Cloud.

**Figure 5:**
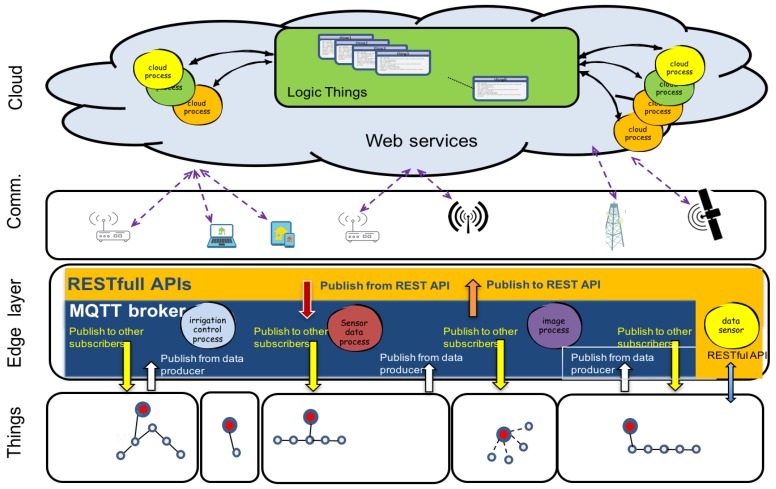
Platform Architecture. Internet and Intranet computing are integrated in the Edge Layer. Local processes pushes applications, data and computing power (services) away from local points to the logical extremes of the network. Logical things are stored and analysed in Cloud Layer.

This model is shown in Figure 5. Critical applications and basic control processes should be installed in edge layer. Web services, HMI interfaces or analytic applications could be installed on internet/intranet cloud. Other extended services and applications are developed using Internet. Things and Cloud services are distributed, Communication and Edge layers provide the resources to integrate and to make them interoperable.

An activity, service or process is formed by things (inputs and outputs distributed) and processing modules that work taking advantage of the communication facilities. Sometimes they can have storage and user interfaces. Both things and processing modules are distributed in local or global networks.

Edge computing provides reliability of response in control processes. Internet Cloud provides accessibilities, storage and analytic resources that can be exploited by users, things or other services.

Agronomists and experts in control and communication must decide what are the cloud applications and processes in edge layer. Edge layer provides interoperability and time-response. Cloud-services provide web services, data storage and analysis.

Software platform is composed by data acquisition, control-communication processes, cloud-services and tools for agronomists.

### 3.4. Integration of Protocols: M2M, Edge Computing and Web Services

Response times and interoperability are required for control processes in PA scenarios. Human interfaces, data access and analytic services are services on Cloud-Computing. Both of the above-mentioned requirements are treated with different protocols: MQTT on control and RESTful on cloud services. PA IoT applications have to be executed in a distributed cloud, instead of in one, big data centre. This will require breaking up large and complex computations into smaller tasks (irrigation, climate, images, crop, energy, water, nutrients) that can be distributed. These smaller tasks make interoperable different data sources and control process algorithms: all these tasks are part of the edge computing layer. In this layer, applications M2M using MQTT protocol, control, data process and data communication to cloud using RESTful protocol are developed.

MQTT uses an open message protocol that enables the transfer of telemetry-style data (i.e., measurements collected in remote locations) in the form of messages from devices and sensors, along unreliable or constrained networks, to a server (BROKER). Messages are simple, a compact binary packet payload (compressed headers, much less verbose than HTTP), and it makes a good fit for simple push messaging scenarios such as temperature updates or mobile notifications. It also works well connecting constrained or smaller devices and sensors to a web service, for example.

**Figure 6:**
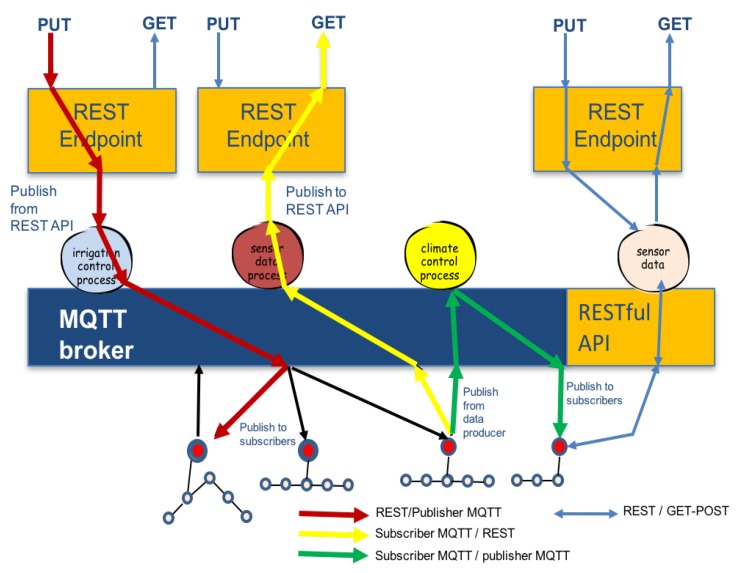
REST/PUT-GET and MQTT subscriber/publisher interactions.

The Publish/Subscribe model used in MQTT and many other M2M systems is mapped to resource observers. PUT and GET operations on HTTP / REST are integrated on MQTT broker server. Publisher and subscribers clients of different networks can acquire or produce data: interoperability between networks is provided. Figure 5 shows multiple processes connected to a BROKER that participate in read-write sharing of REST endpoints across MQTT (or other protocol) connections. Figure 6 shows the potentials operations performed on edge layer that integrates MQTT protocol (used in control processes) and REST/HTTP protocol (used in web services):

- **REST/Publisher MQTT**: shows the Process-Publisher publishing the update resulting from an HTTP PUT to update the resource. In general, a create operation could also result in the publishing of a topic. A Process publishes a topic when an HTTP PUT is received. Examples of this operations can be manual control, rules settings, cloud-services request, etc..
- **Subscriber MQTT/REST**: shows how the Process-Subscriber creates a subscription in the broker, which updates the REST endpoint when a message is published from the broker on the topic corresponding to the resource path. Process performs HTTP GET operation when a topic subscribed is updated.
- **Subscriber MQTT/publisher MQTT**: shows Real-Time operations performed by a control process using only MQTT protocol. MQTT allows devices to send (publish) information about a given topic to a server that functions as an MQTT message broker. The broker then pushes the information out to those clients that have previously been subscribed to the topics. The design principles of MQTT is to minimise network bandwidth and device resource requirements whilst also attempting to ensure reliability and some degree of assurance of delivery. These principles make MQTT a good M2M choice for IoT and mobile apps.
- **REST/GET-POST**: REST API are libraries, resources and specifies the structure of the data that will be exchanged between different devices and the Server-Cloud. Restful API talks HTTP (the same protocol browsers use to communicate with web pages), the standard way to communicate in the World Wide Web. The RESTful API supports four HTTP methods: GET, POST, PUT and DELETE.

## 4. Experimental Prototype

**Figure 7:**
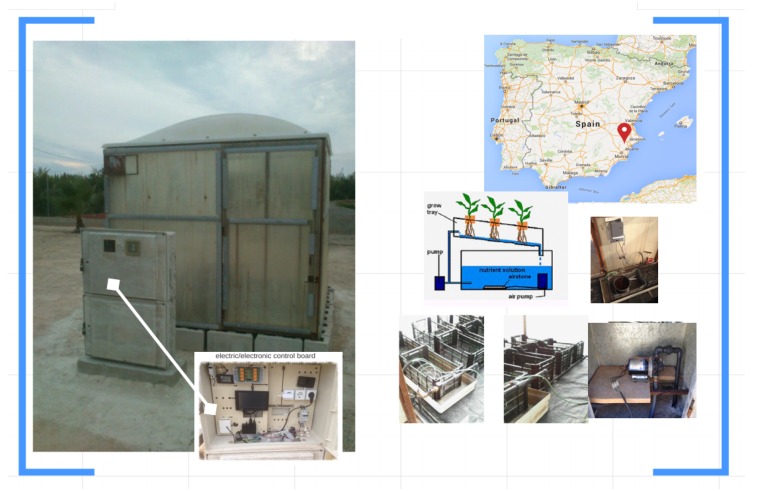
Experimental Hydroponic Station. Hydroponic crop in greenhouse. Localization and different components.

An installation (Figure 7) for hydroponic crop is provided in a green house to design, develop and test the proposed model. Hydroponics is a subset of hydroculture and is a method of growing plants using mineral nutrient solutions, in water, without conventional soil. Terrestrial plants may be grown with their roots in the mineral solution only, or in an inert medium, such as perlite or gravel. Our experimental station uses coco coir as Hydroponic Media (soil).

Requirements of experimental platform (shown in Section 3.1) is also transferred to the specific USN design:

- Low-cost deployment: sensors/actuators (temperature, moisture, PH, EC, Luminosity, electro-valves, pumps, lamps) technology used are not expensive. Controllers and sink nodes (routers) are embedded devices with hardware of widespread use.
- Standard communication protocols and non-proprietary hardware-software. USN networks use WIFI, Bluetooth Low Energy (BLE) and serial bus protocols as support to develop communication services.
- Easy access and maintenance: sensors/actuators and devices are easy to identify, connect and maintain.
- Control processes and edge computing layer development: basic control processes work in local network. Some analytic, storage and user interfaces are distributed in stand-alone cloud and internet services.
- Support for integration of new smart control modules and interoperability: web services protocols (REST, HTTP, MQTT, etc.) and open source hardware-software paradigms allow to program new modules which can be easily integrated. Interoperability between things is assured using IoT applications.
- Provide support for agronomist use, maintenance, settings and basic extensions.
- Analyse agronomist feedback.

In Table 2, things, control processes and cloud services are shown. Agriculture processes are implemented in the edge layer. Applications are installed in different devices that control different sensor network. Using MQTT messages and RESTful operations all sensors/actuators nodes are interoperable. IP addresses in local network, MQTT broker and HTTP/web server allow all control devices to communicate. Control processes are: {imageanalysis}, {nutrientscompositioncontrol}, {light,climatecontrol}, {irrigation,PHandECcontrol}, {energymanagement}, {userinterfaces,datastorage, statisticalcalculations,analytics}.

Each program has logic variables (sensor/actuator things) and rules designed by agronomist. The method and technologies proposed in this work are tested using different type of sensors and embedded devices. Experimental greenhouse has four kind of embedded devices and uses a commercial IoT cloud-services shown in Table 3. The embedded devices are low-cost hardware with adequate computational resources. Devices Type 1 and Type 2 have a cost below 50$. Sensors are standard components that provide an adequate accuracy level, approved by agronomist: moisture, humidity, temperature, luminosity and water flow (4–20 $/sensor), PH and EC sensors (30–70 $/sensor). Similarly, actuators like relay (5–20 $/actuator), led light strip crop (20–50 $/m), water pump (50–100 $/400W), industrial heater (50–100 $/3000W), electro-valve (20–50 $) and router GSM (100–200 $). Magnitude order is about 300–740$ for the entire ubiquitous sensor network platform using Internet of Things.

Three embedded devices control three sensors networks:

1. **USN1.** Monitor water tank using: PH, EC and Temperature sensors. This node control nutrients using electro-valves. This sensor network is managed by an embedded device type 2 (Table 3).
2. **USN2.** Monitor radiance using: radiance and luminosity sensor. Monitor soil and environmental conditions using: temperature, soil moisture, humidity and flow sensors. This node controls the electro-valve irrigation and illumination control relay. This sensor network is managed by an embedded device type 1 (Table 3).
3. **USN3.** Monitor greenhouse climate using: temperature, humidity sensors. Takes images with embedded camera. This node control automated windows and air conditioning system. This sensor network is managed by an embedded device type 1 (Table 3).

### MQTT, Edge Layer and RESTful Integration

**Figure 8:**
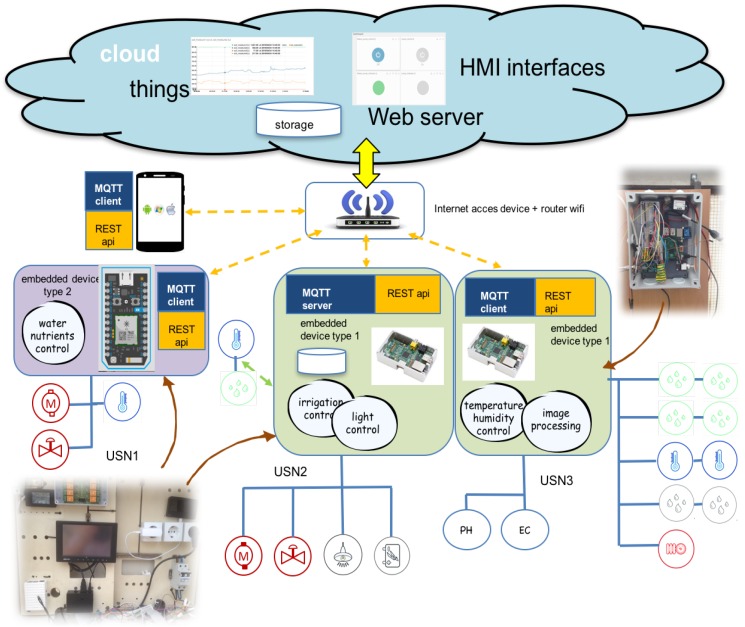
Experimental Hydroponic Station deployment. IoT communication is tested using three USN controlled by two kind of embedded devices. Sensors and actuators are logical variables in Ubidots IoT framework. GUI interfaces, analytic, storage and events programming are tested during plants growth. Control local processes are implemented in these devices.

**Figure 9:**
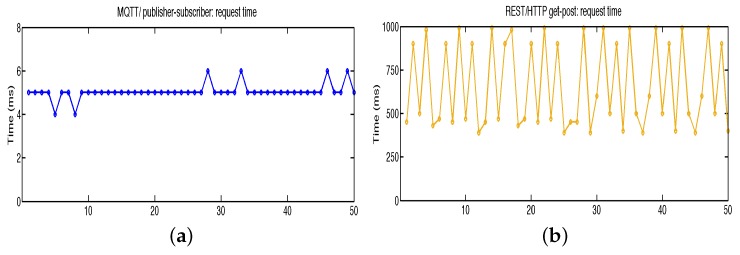
Response time (**a**) Messages using MQTT server on control processes; (**b**) HTTP requests using cloud web server on graphical data monitoring.

Figure 8 shows the deployment of work programs devices and sensor networks on the experimental hydroponic station. Agronomist design rules for control and automated agriculture production. First rules implemented on irrigation, climate and nutrients are developed. MQTT broker is installed in embedded device type 1. Soil temperature installed on USN2 is transmitted to processes on USN1 and USN3 using MQTT broker with Quality of Service (QoS) level 1. This level attempts to guarantee that a message is received at least once by the intended recipient. Once a published message is received and understood by the intended recipient, it acknowledges the message with an acknowledgement message (PUBACK) addressed to the publishing node. Until the PUBACK is received by the publisher, it stores the message and retransmits it periodically. QoS Level 2 attempts to guarantee the message is received and decoded by the intended recipient. This is the most secure and reliable MQTT level of QoS. This type of message may be useful for turning on or off critical control. In this first experience QoS Level 1 is used in switch ON-OFF actuators and publish data sensors. In Figure 9 time response of messages MQTT (a) and data sensor pushed to cloud-IoT platform (b) are tested. The experimental results show the different order of magnitude: 4–6 ms for MQTT messaging and 400–1000 ms for cloud-server access.

The Internet of Things (IoT) exploits network of objects, sensors, and things. Robust and flexible REST API simplifies the storage and retrieval of sensor data across multiple hardware platforms. It can also add any type of metadata and attributes to data sources, like *type: temperature sensor_type: temperature sensor* or *location: greenhouse*, building powerful relationships within the data. These platforms provide support for different languages: python, Java, C, PHP, Node and Ruby are some examples, the aim is to cut down application development time. In this first experience HMI is based in web graphics of data sensors through custom widgets like line charts, gauges, multi-line charts, scatter plots or maps. Agronomist can customize the look of the dashboard through simple drag-n-drop gestures, or changing the size of widgets.

Other cloud-services like Analytic are provided from an arbitrary date range, calculate statistical figures over any time period : mean, max, min, count, sum and variance are numeric calculation that can be performed with the captured dataset. From API authentication, to data traffic and storage, web security is provided. PA data reach cloud services securely thanks to a token-based authentication and an optional HTTPS/SSL encryption layer. IoT platforms push data from any Internet-Enabled Device and prompt them to quickly get started. Similar platforms with similar services show the state of commercial IoT technology: Ubidots [33], Thingspeak [34], Zatar [35], Echelon [36] or Xiveli [37] are some examples of companies that provide IoT services. These platforms are built with similar architectures and provide, usually, the same resources: API REST communication between clients and IoT server. I Python language is used to develop control algorithms designed by the agronomist. REST API Ubidots libraries are integrated in control processes for use in communication operations. Cloud storage of sensor data is obtained in CSV format files. GUI Libraries provide resources to create different graphic interfaces. Events or alarms can be programmed by the agronomist on the cloud. Basic analytic is provided by the platform: raw, average, maximum and minimum.

**Figure 10:**
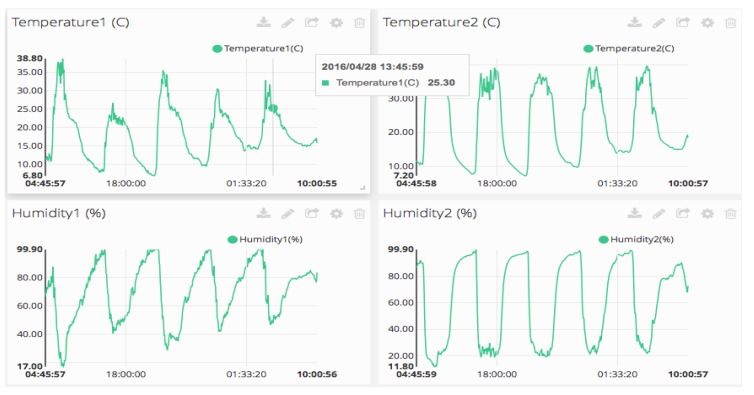
Example of Temperature and relative Humidity of greenhouse (in/out) showed on cloud-web server. Sampling time is defined by the agronomist. These sensors are included in USN3.

Example of a graphic interface, created with Ubidots cloud-platform is shown in Figure 10. Different kinds of graphics can be selected configuring a dashboard easily designed and modified by users.

Table 4 shows different services that can be used like graphics, manual control using dashboard GUI, events that can be programmed: trigger alerts when a sensor value hits a threshold. Easily configure under what conditions an alert should be triggered. Agronomist can set any type of thresholds through simple IF...THEN statements. For example: if a temperature is too low, an event can be programmed using cloud level. A specific action can be executed. In some cases to send an Email or SMS alerting someone about a specific event. Alternatively, it can request a custom URL which can be hooked to an actuator (like an electrovalve) or trigger an action inside another app. Finally, agronomist can create ON/OFF widgets in their dashboard and start controlling things remotely, at the click of a button. Some things (actuators) are controlled using this level. This kind of control must be consistent with automated control implemented on edge layer.

## 5. Results and Conclusions

An hydroponic cultivation has been designed following the method proposed in this work using sensor networks, embedded devices, IoT paradigms and agricultural control processes. The main goal is to exploit the advantages of these technologies in PA installations. Low-cost elements, open systems, reusable applications, easy access, friendly interfaces and interoperability between devices are some of these benefits. A greenhouse station was used to test the growing of a crop using the methods and technologies proposed. The results obtained on the growth plant are shown in Table 5. Using emerging embedded hardware technologies and standard sensor/actuator with adapted accuracy, all components may be replaced by different manufacturers, achieving best price. Open software and internet resources decrease the final price even more when compared to some industrial solutions. The results experienced by the agronomist and users were positive. The system developed provides new ways of access to information which is obtained easily with the communication services. Users can interact with applications through simple conditional statements and quickly adapt to changing processes requirements: the process control can be adapted to real needs in less time. Furthermore, through information provided, water used in greenhouse irrigation process (hydroponic substrate) was compared with the theoretical consumption needed if soil was used. Water consumption in experimental hydroponic measured is lower (20%) than water needed if evapotranspiration (FAO Penman-Monteith method [38]) was considered. Evapotranspiration equation is a reference to calculate the water consumption needs. The agronomists use this information to program irrigation in traditional processes. In the experimental greenhouse, data obtained by different installed sensors (soil moisture, water and soil PH, water and soil EC, water and soil temperature, ambient temperature and relative humidity) may be used to design different rules that control actuators (electro-valves, lights and electric-pumps). These rules are easily adapted to the conditions and evolution of each specific production.

Interoperability has allowed to develop more advanced controls processes. Algorithms adapted to installation characteristics, local climate and expert knowledge in growing crop can be designed and implemented easily and in a shorter space of time.

**Figure 11:**
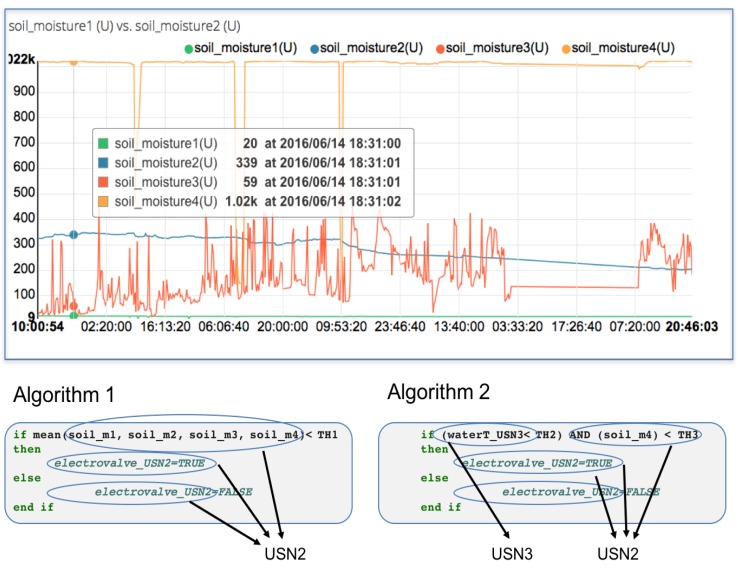
Cloud server graphic of soil moisture sensors data and control algorithms designed by agronomist.

In Figure 11 an example of this potential is shown: Algorithm 1 represents the firsts conditions which controlled irrigation electro-valve. All sensors and actuators belonged to USN2. Experiences with the plant growing and agronomist conclusions modified initial conditions. Algorithm 1 was replaced by Algorithm 2, and in this situation sensors and actuators of USN2 and USN3 were required. Changes in control process and creating new publisher-subscriber messages were performed easily. Feedback from the agronomist is an important issue for a continuous improvement process. New ways to understand processes and usability are some of its most important features. Storage and analytic provide tools to improve growing control. These systems become more efficient and energy management strategies can be developed. The data aggregation service, which collects dataset from the connected things, is another advantage.

Finally, there are no dependencies on proprietary technologies: any sensor, actuator or control device can be integrated. New applications modules or software services are easily designed and implemented. Internet protocols and cloud computing assure the durability of these networks. In the near future, algorithms based in expert systems and artificial intelligence (AI) are planned on this experimental station. Internet Protocol and energy consumption of embedded devices using harvesting resources are another works that will be addressed through IoT services.

New agricultural crops will be soon developed to integrate and test new services using the method and platform developed.

## Figures and Tables

**Table 1:** Related IoT and USN PA works. Main characteristics.

Characteristics	Sensor/Actuator and Control Layer	Communication Layer	Application Layer	Theoretical/Experimental Development
Model for PA on IoT based on the key technologies: Sensors, IoT paradigms, Cloud-Computing, Mobile-Computing and Big-Data analysis [22,23,24]	Different sensors are used: Temperature, Humidity, Soil Moisture, etc. and central server is proposed for data processing	Mobile and Wireless sensor networks like Zigbee, Bluetooth, WIFI. Wired serial bus protocols	Web services: database, data analysis, graphic interfaces	Theoretical models: concepts and architectures. No experiments on real production
Control Agriculture system for production, irrigation, climate, etc. Different technologies are used in the system, such as sensors, RFID, industrial control and so on [25,26]	Deploy different kinds of sensors. Control plant with "IF THEN" rules in embedded devices	Wireless Sensor Network based in nodes Zigbee, 3G and wireless gateway	Cloud services Not deployed	Experimental control and preliminary tests
Monitoring system to analyse crop environment, and the method to improve the efficiency of decision making by analysing statistics. [27,28]	IoT sensors: soil, PH, humidity, temperature, etc.	Wireless gateway and IoT sensor	Monitoring and statistic analysis using internet and web services	Preliminary tests based in monitoring agricultural production and simulation
Scientific or Industrial systems based in integration of internet and web services on automation and industrial control. Proprietary Systems designed for monitoring or large production plants [19,29,30]	Sensors and Industrial control	Ethernet and industrial control protocols, wireless, gateway, modem, etc.	Software control and data acquisition systems (SCADA), Human Machine Interface, web services	Proprietary systems proposed for large production. Ad-hoc systems

**Table 2:** Analysis and design requirements in experimental hydroponic project.

	Analysis and Design Requirements	Things and Control Processes
**crop**	Hydroponically grown plants have the same general requirements as field-grown plants. The major difference is the method by which the plants are supported and the inorganic elements necessary for growth and development are supplied: Temperature, light, water, oxygen, mineral nutrients and support are the control parameters.	sensor/things: {growingcropimages} agricultural-processes: {imageanalysis}
**soil**	In a garden the plant roots are surrounded by soil that supports the growing plant. A hydroponically grown plant must be artificially supported, usually with string or stakes. Soil moisture must be monitored.	sensor/things: {soilmoisture,soiltemperature} agricultural-processes: {nutrientscompositioncontrol}
**climate**	Plants grow well only within a limited temperature range. Temperatures that are too high or too low will result in abnormal development and reduced production. Warm-season vegetables and most flowers grow best between 15 and 25 ÂºC. All vegetable plants and many flowers require large amounts of sunlight. Special plant-growth lamps can be used to grow. Relative humidity should be between upper and lower limits.	sensor/things: {temperat.,luminosity,humidity} actuator/things: {heater,lamp,humidifier} agricultural-processes: {light,climatecontrol}
**water**	A controlled (time and flow) irrigation is necessary. If the aggregate is not kept sufficiently moist, the plant roots will dry out and some will die. PH and Electro-Conductivity (EC) are variables that need to be controlled.	sensor/things: {PH,EC,irrigationflow} actuator/things: {electrovalve,waterpump} agricultural-processes: {irrigation,PHandECcontrol}
**nutrients**	Soil-less growing requires complete and effective hydroponic nutrient solutions. Liquid nutrients (nitrogen, phosphorus, potassium) are prepared by agronomist.	sensor/things: {nitrogen,phosphorus,potassium}
**energy**	Monitoring energy consumption and controlling photovoltaic generation enables powering devices only when needed. The energy balance of the activity (processes and things) must be analysed.	sensor/things: {energymetersensors} actuator/things: {switch,photovoltaicpanel} agricultural-processes: {energymanagement}
**cloud service**	Storage, analytic and user interfaces must be designed. Tables and graphs with statistical data show data in real time. IoT resources store principal data. Subsequent analysis generate information about the growing process	things: {tables,graphs,variables,events} cloud-processes: {userinterfaces,datastorage, statisticalcalculations,analytics}

**Table 3:** Embedded devices used in experimental greenhouse.

	Capabilities			
	Processing	Programming	Communication/Control	Storage
Type1: RaspberryPi 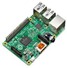	ARM Cortex-A7 CPU 900 MHz Memory 1GB	Linux C/C++ and Python APIs libraries	WIFI and USB SPI and MQTT protocol GPIO and A/D modul	micro SD 16 GB
Type2: Photon IoT 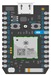	ARM Cortex-M3 CPU 120 MHz 1MB flash, 128KB RAM	C language APIs libraries	WIFI and USB SPI and MQTT protocol GPIO and A/D ports	-
Type 3: SmartPhone 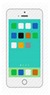	SoC *μ* Processor CPU ≥ 900 MHz Memory ≥ 1GB	Android/iOS Objective C Java libraries	WIFI and USB Bluetooth 4G LTE	-
Type 4: WIFI router 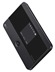	SoC *μ* Processor	-	WIFI Bluetooth 4G LTE	-
Cloud-server: IoT ubidots 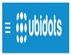	Push Data from any Internet-Enabled Device	REST API HTTP request Python, Java, C, PHP, Node and Ruby lib.	Internet connection Analysis and visualisation Trigger actions: e-mail, SMS and WebHook	Data storage

**Table 4:** Main Resources, services and information examples provided by IoT platform (Ubidots).

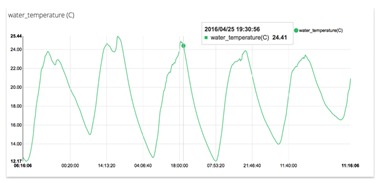	Water temperature graphic obtained. This sensor is included in USN1 and embedded device type 2. This chart is useful in water nutrient process.
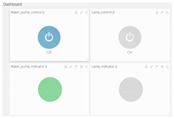	Control dashboard and state indicator. These actuators are included in USN3 (manual control). Automatic control is implemented in embedded device type 1.
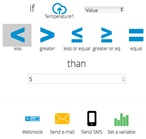	Events with trigger actions can be programmed by agronomist. An SMS can be mailed when data sensor reaches a fixed value. E-mail also can be used.

**Table 5:** Growth crop process on experimental station. Beans Growth stages.

Days 0–10 vegetative stage	Days 10–15 emergence stage	Days 15–2 cotyledon	Days 20–25 unifoliolate nodes
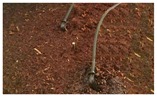	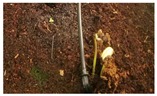	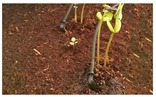	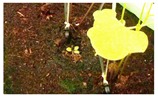
Irrigation actual (Total liters) = 20 L/m${}^{2}$		Average Temperature = $15{\;}^{\circ }$C	
Irrigation theoretic (Total liters **) = 25 L/m${}^{2}$		Average water PH = 6.5	
** Evapo-transpiration (ETP) FAO equation		Average water EC = 1000 μS/cm	
Energy used = 60 Wh/m${}^{2}$/day		Solar irradiance = 4 kWh/m${}^{2}$/day (NASA HOMER web)	
**Reduction in water consumption = 20%**			
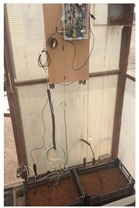	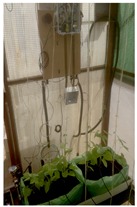	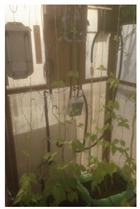	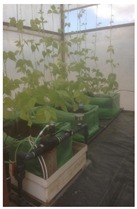
**Problems**	**Solutions**		**Technological results**
Initial expectations unfulfilled Complexity of technology Incompatibility of components Lack of products High costs	Develop a reference model based in IoT paradigms and USN resources Standard components (devices, sensors, actuators) with low cost PA processes automated with software and languages standard Graphics Interfaces use simple and universal access Tools, facilities and resources create for agronomist and users needs New products are created integrating technological resources		GUI interfaces used on Internet New ways of data access Low-cost deployment Users can interact and created conditional statements PA information in near real time
